# Deciphering the Multifactorial Susceptibility of Mucosal Junction Cells to HPV Infection and Related Carcinogenesis

**DOI:** 10.3390/v9040085

**Published:** 2017-04-20

**Authors:** Michael Herfs, Thing R. Soong, Philippe Delvenne, Christopher P. Crum

**Affiliations:** 1 Laboratory of Experimental Pathology, GIGA-Cancer, University of Liege, 4000 Liege, Belgium; P.Delvenne@ulg.ac.be; 2 Division of Women’s and Perinatal Pathology, Department of Pathology, Brigham and Women’s Hospital, Harvard Medical School, Boston, MA 02115, USA; tsoong@partners.org (T.R.S.); ccrum@bwh.harvard.edu (C.P.C.)

**Keywords:** human papillomavirus, squamo-columnar junctions, (pre)neoplastic lesions

## Abstract

Human papillomavirus (HPV)-induced neoplasms have long been considered to originate from viral infection of the basal cell layer of the squamous mucosa. However, this paradigm has been recently undermined by accumulating data supporting the critical role of a discrete population of squamo-columnar (SC) junction cells in the pathogenesis of cervical (pre)cancers. The present review summarizes the current knowledge on junctional cells, discusses their high vulnerability to HPV infection, and stresses the potential clinical/translational value of the novel dualistic model of HPV-related carcinogenesis.

## 1. Introduction

Cancer of the uterine cervix is generally observed near the squamo-columnar (SC) junction and is almost always caused by carcinogenic human papillomaviruses (HPV). However, the nature of cervical epithelial cells initially infected by HPV has been a matter of debate/speculation for a long time. Classically, the HPV-target cells are presumed to be the basal keratinocytes of the pluristratified mucosa lining the outer part of the cervix (ectocervix) and the region where the endocervical columnar epithelium, sensitive to the acidic vaginal pH, has undergone a squamous metaplastic transformation during puberty (transformation zone (TZ)). According to this theory, microtrauma, ulcerative lesions, or abrasions into the squamous epithelium would render the proliferating basal cell layer uniquely vulnerable to viral infection [1]. This hypothesis was emphasized by (1) the detection of HPV transcripts in basal cells; (2) the basal location of dysplastic cells in the initial phase of malignancy (low-grade cervical intraepithelial neoplasia (CIN1)); and (3) the observation that chemical disruption of the integrity of the stratified epithelium is required for pseudovirion infection of the murine genital tract [2]. Obviously, it is indisputable that transcriptionally-active HPV infection can occur in basal keratinocytes and result in the subsequent development of (pre)neoplastic lesions in both the ectocervix and the lower genital tract (vagina/vulva). This mechanism is also likely to explain the HPV infections occurring in both the oral cavity and the anal margin/perianal skin (microtrauma resulting from mastication and anal intercourse, respectively) because these two sites are entirely lined by a pluristratified epithelium. However, this hypothesis is incompatible with the historical observation that HPV infection causes cervical cancer and its precursor lesions mainly within the SC junction microenvironment [3,4]. In addition, this theory does not explain the discrepancy in terms of cancer risk between the uterine cervix and other HPV-infected mucosa. Indeed, with approximately 520,000 new cases diagnosed each year worldwide, cervical squamous cell carcinoma (SCC) is approximately 20-fold more prevalent than vaginal/vulvar/penile neoplasms [5]. Finally, considering that routine cervical screening allows for the annual detection of several millions of CIN, no biological or physical factor is likely to induce traumatic lesions in the cervical os of such a large number of women.

In the early 2000s, endocervical reserve cells were also proposed as the cell of origin for cervical cancer [6]. However, the broad distribution of reserve cells in the cervical canal [7], the extremely low reported incidence (less than 1/1000) of CIN in endocervical polyps [8], and the absence of convincing evidence demonstrating HPV infection in normal monolayered reserve cells do not support a major role of these latter cells in cervical carcinogenesis.

**Figure 1:**
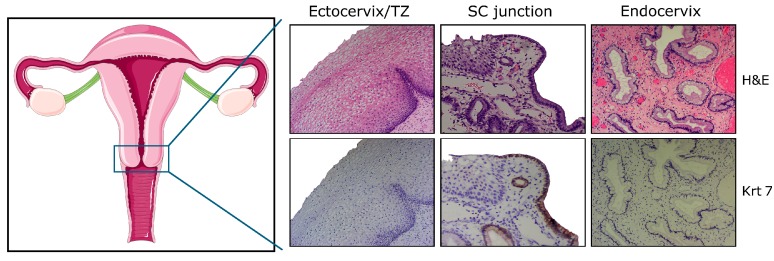
Schematic representation of the female genital tract and histology of the adult cervix with ectocervical (squamous), junctional (cuboidal), and endocervical (columnar) cells. Note the uniform keratin 7 (Krt7) immunoreactivity displayed by cuboidal cells observed within, or in close proximity to, the squamo-columnar (SC) junction. H&E: hematoxylin and eosin; TZ: transformation zone.

Consequently, for years, it has been thought that the ecto-endocervical junction microenvironment contains unique epithelial cells highly susceptible to high-risk HPV infection and related (pre)cancer development. However, the topographical preference of cervical neoplasia for the SC junction remained unexplained. In 2012, a discrete population of cells residing within, or in close proximity to, the cervical SC junction was identified [9] (Figure 1). Displaying a cuboidal/immature columnar phenotype, these cells were shown to have an embryonic origin similar to their counterparts discovered one year earlier at the gastroesophageal junction [10,11]. In addition to being actively involved in adult adaptive processes (metaplasia, hyperplasia) [10], SC junction cells exhibit intriguing phenotypic similarities with approximately 90% of cervical SCC and high-grade precursors [9,12]. These findings underlying the instrumental role of junctional cells in cervical carcinogenesis were recently confirmed by several immunohistochemical and/or transcriptional studies analyzing the expression of SC junction-overexpressed biomarkers (i.e., cytokeratin 7 (Krt7), anterior gradient protein 2 (AGR2) or cystic fibrosis transmembrane conductance regulator (CFTR)) in large cohorts of cervical (pre)neoplastic lesions [13,14,15,16,17]. Moreover, the evidence that normal-appearing SC junction cells harbor both HPV transcripts (E6*I/II) and early viral proteins (HPV E2) was reported and sustains the possibility that these cuboidal cells could serve as a reservoir for latent infections and subsequent CIN development in asymptomatic HPV-positive patients [18].

## 2. High Vulnerability of SC Junctions to HPV Infection and (Pre)Cancer Development: A Multifaceted Process

### 2.1. Altered Secretion of Antimicrobial Peptides

Similar to the skin or other mucosal surfaces, the gynecological tract expresses a distinct set of host–defense peptides adapted specifically for limiting pathogen invasion and replication within this unique environment [19]. Involved in several processes beyond their antimicrobial activity (i.e., wound healing, angiogenesis, etc.) [20,21], several innate peptides, especially the lysozyme, the secretory leukocyte protease inhibitor (SLPI), as well as the defensin superfamily, have been recently the subject of extensive mechanistic studies. Using pseudoviruses of both cutaneous and mucosal (non-carcinogenic (low-risk) and carcinogenic (high-risk)) genotypes, Buck and colleagues first demonstrated that alpha defensins 1–3 (also called human neutrophil peptides (HNP) 1–3) and 5 (known as human defensin 5 (HD5)) strongly inhibit HPV infection in vitro [22]. In contrast, beta defensins, lyzosyme, or SLPI exhibited very little or no antagonist activity. Based on these important findings and the strong chemotactic activity exerted by alpha defensins for antigen presenting cells, these latter peptides were considered as interesting candidates for the non-surgical management of HPV-positive lesions in young women [23]. Despite encouraging results [24], the therapeutic effect of these antimicrobial peptides was never precisely determined in vivo. Recently, interest in the defensin superfamily in the context of HPV infection was reactivated by the observed absence of HD5 expression in both normal SC junction cells and (pre)neoplastic lesions arising from these latter cells [25]. Moreover, the HD5-related anti-HPV activity was recently deciphered. By preventing proteolytic processing (furin cleavage) of the minor capsid protein L2, Wiens and Smith showed that HD5 does not block virus internalization, but significantly alters the viral entry pathway [26]. The same research team further investigated the molecular mechanism and demonstrated that, similarly to the inhibitory effect reported for both human adenoviruses and polyomaviruses [27,28,29], HD5 alters HPV intracellular trafficking via capsid stabilization and redirection of the incoming viral particles to the lysosome [30]. Altogether, these data suggest that SC junction cells may display an increased vulnerability to HPV infection through a deficient expression of innate molecules inhibiting the intracellular steps of virus processing.

### 2.2. Overexpression of Key Proteins Implicated in Post-Endocytic HPV Trafficking

Although some unclear points remain (i.e., the identity of the internalization receptor(s) or the pathway(s) involved in HPV entry), the mechanisms governing HPV entry into host cells is now relatively well-characterized [31]. After initial binding to heparan sulfate proteoglycans, several conformational changes and, as mentioned above, a furin-dependent L2 cleavage have been described. Viral particles would then interact with a complex of proteins including alpha 6 integrin and tetraspanin CD151 before their endocytosis in a clathrin- and caveolin-independent manner. Following HPV entry, virions are transported via the endosomal system where capsid disassembly occurs. Significantly, a recent article demonstrated the crucial requirement of tetraspanin CD63, a SC junction-overexpressed protein [9], during post-endocytic steps [32]. Accordingly, CD63 was shown to interact with L1 capsid protein and virus uncoating was dramatically decreased in CD63-depleted cells. In contrast, this junctional-overexpressed molecule was not involved in cell surface interactions or endocytosis. Given that post-endocytic trafficking strongly determines the success of the infection, the high expression of some key proteins controlling both vesicular trafficking and HPV disassembly could render the SC junction cells more vulnerable to HPV infection.

### 2.3. Possible Translational Regulation of HPV mRNAs by the Cytokeratin Filaments 7 and 19

All mucosal HPVs are characterized by a circular DNA genome of approximately 8000 base pairs coding for eight major proteins (early region: E1, E2, E4, E5, E6, E7; late region: L1 and L2). Viral gene expression has been shown to be regulated at the level of transcription (by HPV E2 protein [33], as well as host cell factors, such as Specificity protein 1 (Sp1) and Activator protein 1 (AP-1) [34]), polyadenylation, and RNA splicing. The latter is evidenced by the full transcriptional maps reported for several HPV genotypes [35,36,37,38]. In the early 2000s, several studies analyzed the interactions occurring between viral transcripts and intermediate filaments and reported exciting effects of Krt7 and 19 on E7 oncoprotein expression. Indeed, by interacting with a 6-mer amino acid peptide SEQIKA present at position 91–96 in the human Krt7 sequence, HPV E7 messenger RNA (mRNA) was shown to be protected/stabilized [39]. In addition, viral mRNA translation was presumed to be increased by Krt19 [40], another member of the keratin family overexpressed in both cervical and anal SC junctions [17,41]. Although these intriguing findings need to be confirmed, they support the hypothesis of a specific regulation of viral transcripts in SC junction cells leading to increased viral oncoprotein levels and, subsequently, to (pre)cancer development/progression.

### 2.4. Altered Adaptive Immune Responses in SC Junction Microenvironment

The junction between the ectocervical squamous and the endocervical muco-secreting epithelia appears to be dynamic. Indeed, this junction moves to the ectocervix until the age of about 14. Then, a metaplastic process occurs due to an estrogen-dependent acidification of the vaginal luminal pH and this subsequent replacement of the columnar epithelium by metaplastic squamous foci traces the SC junction into the endocervical canal, where it is often highly situated in menopause. The regenerative metaplastic response (TZ) between the former and the new SC junction has been shown to be driven by the junctional cell-dependent production of squamous metaplastic (reserve) cells [10]. In general, the metaplastic epithelium does not exhibit the same structure throughout the transformation zone. Depending on the persistence, or not (exfoliation), of the remaining precursor SC junction cells at the top of the metaplastic epithelium, the degree of metaplasia is considered as immature or mature, respectively. This histological feature is probably related to vaginal pH fluctuations during the menstrual cycle as, in healthy women, pH ranges between 4.5 and 5.5 with alkalinization before ovulation [42]. Therefore, a chronic, but inconstant, irritation of the mucosa occurs inducing a chronic inflammation in the cervical os.

**Figure 2:**
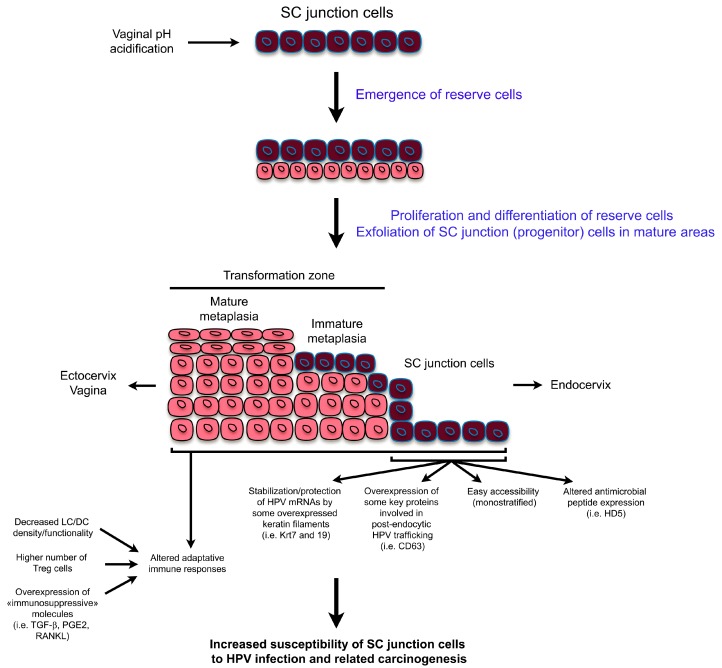
Schematic representation highlighting both the tissue remodeling observed in the SC junction microenvironment and the possible mechanisms explaining the high susceptibility of the SC junction cells to human papillomavirus (HPV)infection and related carcinogenesis. mRNA: messenger RNA; HD5: human defensin 5; LC/DC: langerhans cells/dendritic cells; PGE2: prostaglandin E2; RANKL: receptor activator of nuclear factor κ-B ligand.

Prior to and following the discovery of SC junction cells, we, and others, reported altered local immune responses within the SC junction or in metaplastic areas (TZ) located in close proximity. Focusing on CD1a^+^ antigen-presenting cells (Langerhans cells (LC) and dendritic cells (DC)), several studies showed that LC/DC density is largely decreased in both the SC junction microenvironment and the cervical (pre)neoplastic lesions compared to the surrounding HPV-uninfected ectocervical/vaginal squamous mucosa [43,44,45]. By inducing the expression of epithelial to mesenchymal transition regulators, the strong secretion of transforming growth factor- β (TGF-β) observed in TZ (especially in immature metaplastic patches) was shown to inhibit E-cadherin expression [43]. The resulting disruption of E-cadherin-mediated LC-epithelial cell adhesion was shown to alter antigen presenting cell maturation/differentiation promoting T regulatory (Treg) cell development [46,47]. Moreover, an inverse correlation between LC/DC density and the expression of prostaglandin E2 (PGE2) enzymatic pathways was reported [48]. The significance of these observations in terms of antigen presenting cell trafficking was clearly demonstrated. Indeed, PGE2 decreased the migratory capacity of immature LC/DC as well as induced the tolerogenic phenotype of these cells by altering accessory molecule expression and by modifying Interleukin (IL)-10/IL-12 secretion ratio [48]. Similarly, receptor activator of nuclear factor κ-B ligand (RANKL), which is strongly secreted in both the cervical SC junction and HPV-related (pre)neoplastic microenvironment, was also shown to promote the emergence of tolerogenic DC [49]. The altered density of LC/DC within, or in close proximity of, the SC junction could be further exacerbated by the absence of HD5 expression, which has been shown to have a strong chemotactic activity on several cell types involved in the immune responses [25]. In addition to LC/DC, the functionality of both T lymphocytes (higher number of Treg cells) and plasmacytoid dendritic cells (pDC) is also altered within the cervical SC junction microenvironment and the high mobility group box 1 (HMGB1) was recently identified as a key soluble factor involved in the acquisition of tolerogenic pDC [50]. Altogether, the modified expression of soluble/adhesion molecules within the SC junction microenvironment could promote the immunoescape of infected/transformed cells. The possible mechanisms underlying the mucosal junctional cells’ unique susceptibility to HPV-related carcinogenesis are summarized in Figure 2.

## 3. Dualistic Model of HPV-Related Carcinogenesis

### 3.1. Explanation for Multiple Neoplastic Phenotypes

In pathology practical examination, it is well established that HPV genotypes impact the morphology of (pre)neoplastic lesions. Low-risk HPVs (mainly HPV6 and 11) are typically related to condylomata acuminata (genital warts) development and viral cytopathic effect [51,52]. In contrast, carcinogenic (high-risk) strains are less frequently associated to koilocytic morphology or virion production [52,53]. In addition to the viral component, recent findings support that the nature of the epithelial cells (basal keratinocytes versus SC junction cells) originally infected by HPV also considerably influences the appearance of CIN. While the infections occurring in the ectocervix (and, in general, in the lower genital tract) lead, traditionally, to well-differentiated squamous lesions, an immature phenotype is frequently observed when high-risk HPV-mediated transformation appears within the endocervical canal [12]. These morphological differences governed by the expression profile/intrinsic features of “the cell of origin” critically affect the pathologists’ interpretation and subsequent classification of HPV-related lesions. Indeed, diagnostic reproducibility of CIN is well-known to be problematic leading to inappropriate management of patients [54]. The inter-observer agreement is especially low (~50%) for Krt7-positive low-grade dysplasias (arising from the SC junction) and CIN2 supporting that this latter “considered high-risk” lesion cannot be consistently distinguished in the spectrum of cervical (pre)cancers [12,55]. This has led to proposals that this uncertainty be conveyed in the diagnostic report (i.e., (pre)neoplastic lesions of uncertain grade (CIN1-2)) and managed accordingly [56]. 

**Figure 3:**
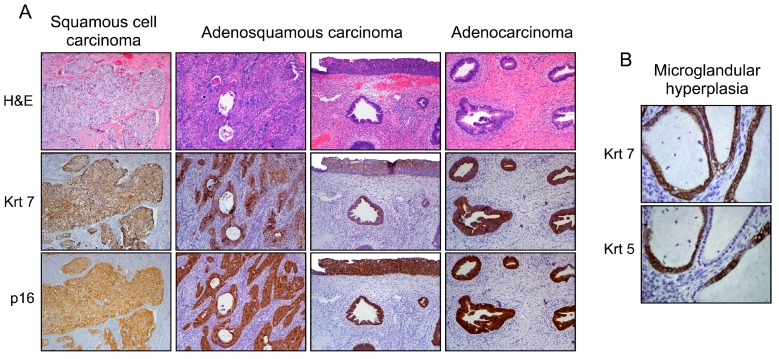
Phenotypic variants among cervical malignant epithelial tumors (**A**). Note the Krt7 immunoreactivity displayed by squamous, adenosquamous (blended or individuated/adjacent lesions), and columnar neoplasms supporting their similar cell of origin (SC junction); and (**B**) under benign conditions (i.e., microglandular hyperplasia), a mixed phenotype can also be observed. Note the Krt5 (squamous biomarker) expression in Krt7-positive cuboidal cells without evidence of reserve cells.

In addition to lesions demonstrating a squamous phenotype (~85% of all cervical neoplasms), adenocarcinoma, as well as intriguing lesions that display both squamous and columnar differentiation are observed within the SC junction microenvironment [57,58,59]. These latter may range from individuated and adjacent squamous (CIN) and columnar (adenocarcinoma in situ) lesions to cases in which both components are closely blended. Such a mixed phenotype can also be detected in benign lesions, such as microglandular hyperplasia [10,60] (Figure 3). Although both the stemness properties and differentiation potential of SC junction cells are still not clearly defined, the expression of SC junction-specific/overexpressed biomarkers exhibited by all types of cervical lesions (squamous, columnar, adenosquamous) represents the strongest argument for the multipotent potential of junctional cells. The immuno-phenotypic homology between cervical SC junction cells observed in adult tissues and embryonic Müllerian epithelium also supports this innovative hypothesis [10].

### 3.2. From the Bench to Cervical Cancer Prevention

Although bi- or quadrivalent HPV vaccines have been on the market for a decade and have been shown to be highly efficient to prevent HPV16/18-related high-grade anal/cervical lesions [61,62,63,64], HPV is still the most common sexually transmitted infection worldwide according to the World Health Organization (WHO). Based on estimations, up to 500 million individuals could be carriers of HPV DNA [65]. Although the large majority of infected patients are asymptomatic and will never develop cancer, every year, billions of euros/dollars are spent for both screening (Pap smear and/or HPV testing) and follow-up of patients at very low-risk of tumor development [66]. In an era of personalized medicine, the identification of one or, more realistically, several reliable biomarkers (used in combination) and allowing to accurately predict the outcome of HPV-related lesions, might substantially relieve the current ponderous and costly management of infected patients [67]. In the last decade, this area of research has been the subject of numerous studies and several candidate (viral and cellular) biomarkers have been highlighted. Among these latter, p16^ink4^, the HPV viral load and genotyping were the most investigated. Although, all gave some indications on high-risk patients, a lack of concordance has been extensively reported and all failed to precisely predict CIN, which will finally progress to cancer [68,69,70,71,72,73,74,75].

Supported by the very large discrepancy, in terms of cancer risk, between the uterine cervix and the vagina/vulva, it is now very likely that the nature of the HPV-infected cells strongly influences disease outcome. Recently, four studies analyzed the predictive value of junctional biomarkers (especially Krt7) and, interestingly, all showed that low-grade CIN arising from the SC junction have a significantly higher risk to progress to CIN2/3 compared to their counterparts observed in the transformation zone/ectocervix [12,13,14,15]. Furthermore, when compared to other risk factors, such as HPV16 infection and diffuse p16^ink4^ expression, full-thickness Krt7 immunoreactivity demonstrated the highest correlation with lesion progression [14]. Although these conclusions were shown to be reproducible and in agreement with the high percentage (~90%) of CIN3 and cervical cancers displaying immunophenotypic similarities with SC junction cells, unfortunately, Krt7 or another SC junction-specific/overexpressed protein is unlikely to be sufficiently specific to provide actionable information in the clinical setting. Indeed, whatever the HPV genotype or the cellular origin of (pre)neoplastic lesions, the majority of HPV infections clear spontaneously (without any medical action) within 1–2 year(s) [76,77]. In addition, predicting disease progression is extremely challenging and subject to several biases, such as the tissue sampling, over/underdiagnosis, and the age of patients. Therefore, despite cumulative efforts for discovering reliable predictive biomarkers, the most cost-effective management of HPV-related lesions is to avoid relying on these costly and imprecise “false prophets”.

HPV vaccines, recommended for young sexually-naive women, hold promise to make a major impact on cervical cancer mortality in the future. However, a large number of older and/or vulnerable women are in need of a cancer preventive. The discovery of SC junction cells might point the way to a different, low-cost, and simple way to prevent cervical cancer in underserved populations: by removing the vulnerable cells that are its source. This recently-proposed clinical perspective is supported by historical data [78,79,80]. Over 50 years ago, Paul Younge (Boston Lying-in Hospital) routinely cauterized the uterine cervices of his patients postpartum. He apparently treated approximately 6000 cervices in this manner and had never witnessed a subsequent cancer in his patients [81]. Several years later, another study noted a profound reduction in cancer risk with cauterization of the cervix [82]. These single practice results were further confirmed by a population-based program conducted in Finland during the 1960s and 1970s [83]. A recent study in South Africa analyzed the occurrence of HPV infections following cervical cryotherapy in healthy (human immunodeficiency virus (HIV)/HPV negative) women. Importantly, a 55% reduction rate was detected in the treated group compared to control individuals [84]. Although prophylactic SC junction (cryo)ablation is unlikely to completely prevent the development of cervical (pre)cancers, this procedure could significantly reduce the risk of subsequent (pre)cancerous lesions similar to what is extensively reported in women who underwent conization [85]. Indeed, these latter patients are characterized by an impressively low recurrence rate given the risk of reinfection by new HPV genotypes, suggesting that the SC junction excision induces a protective effect extending the type-specific immunity potentially acquired by these women. However, this concept still needs to be validated in a controlled clinical trial. The potential value of SC junction ablation in lowering the risk of CIN2/3 in HPV-positive women is another concept worthy of exploration. Repetitive HPV testing is highly inefficient and prone to the vagaries of patient attitudes towards return visits. A strategy in which a single positive HPV test would generate an SC junction intervention of low morbidity might be an interesting alternative in cancer prevention. In addition, by reducing the rate of HPV infections, SC junction removal might also impact on HIV acquisition, which has been shown to be higher in HPV-positive individuals [86,87,88,89,90].

## 4. Conclusions and Perspectives

With the development and commercialization of two vaccines, as well as the Nobel Prize attributed to Harald Zur Hausen, experimental research on HPV-related carcinogenesis was supposed to be (almost) over in the late 2000s. Three major findings/confirmations have recently raised the interest of the whole HPV community: (1) the involvement of beta HPV genotypes in skin cancer development [91,92]; (2) the significance of HPV variants in terms of cancer risk [93]; and (3) the detection of discrete cell populations topographically located in SC junctions and displaying a high susceptibility to HPV infection [9]. The existence of these latter non-squamous cells was assumed for a long time due, mainly, to the observation of most cervical (pre)cancers in the SC junction, as well as the high percentage (~80%) of columnar neoplasms (adenocarcinoma) etiologically linked to carcinogenic HPV [3,4,94]. However, these well-described results were frequently not taken into account by both virologists and epidemiologists. Moving forward, there is little doubt that additional features explaining the high vulnerability of SC junction cells to HPV infection and related carcinogenesis will be discovered in the early future. Therefore, the description of results highlighted in the present review is likely to be incomplete. One additional matter of investigations is the HPV life cycle in SC junction cells and/or adenocarcinoma. Still unknown in these latter cases, it is undoubted that both squamous differentiation and cellular stratification, considered as crucial for the occurrence of a productive infection [95], will not play a role.

## References

[1] Woodman C.B., Collins S.I., Young L.S. (2007). The natural history of cervical HPV infection: Unresolved issues. Nat. Rev. Cancer.

[2] Roberts J.N., Buck C.B., Thompson C.D., Kines R., Bernardo M., Choyke P.L., Lowy D.R., Schiller J.T. (2007). Genital transmission of HPV in a mouse model is potentiated by nonoxynol-9 and inhibited by carrageenan. Nat. Med..

[3] Marsh M. (1956). Original site of cervical carcinoma; topographical relationship of carcinoma of the cervix to the external os and to the squamocolumnar junction. Obstet. Gynecol..

[4] Richart R.M. (1973). Cervical intraepithelial neoplasia. Pathol. Ann..

[5] Forman D., de Martel C., Lacey C.J., Soerjomataram I., Lortet-Tieulent J., Bruni L., Vignat J., Ferlay J., Bray F., Plummer M., Franceschi S. (2012). Global burden of human papillomavirus and related diseases. Vaccine.

[6] Martens J.E., Arends J., Van der Linden P.J., De Boer B.A., Helmerhorst T.J. (2004). Cytokeratin 17 and p63 are markers of the HPV target cell, the cervical stem cell. Anticancer Res..

[7] Martens J.E., Smedts F.M., Ploeger D., Helmerhorst T.J., Ramaekers F.C., Arends J.W., Hopman A.H. (2009). Distribution pattern and marker profile show two subpopulations of reserve cells in the endocervical canal. Int. J. Gynecol. Pathol..

[8] Chin N., Platt A.B., Nuovo G.J. (2008). Squamous intraepithelial lesions arising in benign endocervical polyps: A report of 9 cases with correlation to the Pap smears, HPV analysis, and immunoprofile. Int. J. Gynecol. Pathol..

[9] Herfs M., Yamamoto Y., Laury A., Wang X., Nucci M.R., McLaughlin-Drubin M.E., Munger K., Feldman S., McKeon F.D., Xian W. (2012). A discrete population of squamocolumnar junction cells implicated in the pathogenesis of cervical cancer. Proc. Natl. Acad. Sci. USA.

[10] Herfs M., Vargas S.O., Yamamoto Y., Howitt B.E., Nucci M.R., Hornick J.L., McKeon F.D., Xian W., Crum C.P. (2013). A novel blueprint for ‘top down’ differentiation defines the cervical squamocolumnar junction during development, reproductive life, and neoplasia. J. Pathol..

[11] Wang X., Ouyang H., Yamamoto Y., Kumar P.A., Wei T.S., Dagher R., Vincent M., Lu X., Bellizzi A.M., Ho K.Y. (2011). Residual embryonic cells as precursors of a Barrett’s-like metaplasia. Cell.

[12] Herfs M., Parra-Herran C., Howitt B.E., Laury A.R., Nucci M.R., Feldman S., Jimenez C.A., McKeon F.D., Xian W., Crum C.P. (2013). Cervical squamocolumnar junction-specific markers define distinct, clinically relevant subsets of low-grade squamous intraepithelial lesions. Am. J. Surg. Pathol..

[13] Paquette C., Mills A.M., Stoler M.H. (2016). Predictive Value of Cytokeratin 7 Immunohistochemistry in Cervical Low-grade Squamous Intraepithelial Lesion as a Marker for Risk of Progression to a High-grade Lesion. Am. J. Surg. Pathol..

[14] Mills A.M., Paquette C., Terzic T., Castle P.E., Stoler M.H. (2017). CK7 Immunohistochemistry as a Predictor of CIN1 Progression: A Retrospective Study of Patients From the Quadrivalent HPV Vaccine Trials. Am. J. Surg. Pathol..

[15] Huang E.C., Tomic M.M., Hanamornroongruang S., Meserve E.E., Herfs M., Crum C.P. (2016). p16ink4 and cytokeratin 7 immunostaining in predicting HSIL outcome for low-grade squamous intraepithelial lesions: A case series, literature review and commentary. Mod. Pathol..

[16] Peng X., Wu Z., Yu L., Li J., Xu W., Chan H.C., Zhang Y., Hu L. (2012). Overexpression of cystic fibrosis transmembrane conductance regulator (CFTR) is associated with human cervical cancer malignancy, progression and prognosis. Gynecol. Oncol..

[17] Lee H., Lee H., Cho Y.K. (2017). Cytokeratin7 and cytokeratin19 expression in high grade cervical intraepithelial neoplasm and squamous cell carcinoma and their possible association in cervical carcinogenesis. Diagn. Pathol..

[18] Mirkovic J., Howitt B.E., Roncarati P., Demoulin S., Suarez-Carmona M., Hubert P., McKeon F.D., Xian W., Li A., Delvenne P. (2015). Carcinogenic HPV infection in the cervical squamo-columnar junction. J. Pathol..

[19] Yarbrough V.L., Winkle S., Herbst-Kralovetz M.M. (2015). Antimicrobial peptides in the female reproductive tract: A critical component of the mucosal immune barrier with physiological and clinical implications. Hum. Reprod. Updat..

[20] Suarez-Carmona M., Hubert P., Delvenne P., Herfs M. (2015). Defensins: “Simple” antimicrobial peptides or broad-spectrum molecules?. Cytokine Growth Factor Rev..

[21] Hancock R.E., Haney E.F., Gill E.E. (2016). The immunology of host defence peptides: Beyond antimicrobial activity. Nat. Rev. Immunol..

[22] Buck C.B., Day P.M., Thompson C.D., Lubkowski J., Lu W., Lowy D.R., Schiller J.T. (2006). Human α-defensins block papillomavirus infection. Proc. Natl. Acad. Sci. USA.

[23] Hubert P., Herman L., Maillard C., Caberg J.H., Nikkels A., Pierard G., Foidart J.M., Noel A., Boniver J., Delvenne P. (2007). Defensins induce the recruitment of dendritic cells in cervical human papillomavirus-associated (pre)neoplastic lesions formed in vitro and transplanted in vivo. FASEB J..

[24] Zhao S., Zhou H.Y., Li H., Yi T., Zhao X. (2015). The therapeutic impact of HNP-1 in condyloma acuminatum. Int. J. Dermatol..

[25] Hubert P., Herman L., Roncarati P., Maillard C., Renoux V., Demoulin S., Erpicum C., Foidart J.M., Boniver J., Noel A. (2014). Altered α-defensin 5 expression in cervical squamocolumnar junction: Implication in the formation of a viral/tumour-permissive microenvironment. J. Pathol..

[26] Wiens M.E., Smith J.G. (2015). α-defensin HD5 inhibits furin cleavage of human papillomavirus 16 L2 to block infection. J. Virol..

[27] Nguyen E.K., Nemerow G.R., Smith J.G. (2010). Direct evidence from single-cell analysis that human α-defensins block adenovirus uncoating to neutralize infection. J. Virol..

[28] Zins S.R., Nelson C.D., Maginnis M.S., Banerjee R., O’Hara B.A., Atwood W.J. (2014). The human α-defensin HD5 neutralizes JC polyomavirus infection by reducing endoplasmic reticulum traffic and stabilizing the viral capsid. J. Virol..

[29] Smith J.G., Nemerow G.R. (2008). Mechanism of adenovirus neutralization by Human α-defensins. Cell Host Microbe.

[30] Wiens M.E., Smith J.G. (2017). α-Defensin HD5 Inhibits Human Papillomavirus 16 Infection via Capsid Stabilization and Redirection to the Lysosome. mBio.

[31] Day P.M., Schelhaas M. (2014). Concepts of papillomavirus entry into host cells. Curr. Opin. Virol..

[32] Grassel L., Fast L.A., Scheffer K.D., Boukhallouk F., Spoden G.A., Tenzer S., Boller K., Bago R., Rajesh S., Overduin M. (2016). The CD63-Syntenin-1 Complex Controls Post-Endocytic Trafficking of Oncogenic Human Papillomaviruses. Sci. Rep..

[33] McBride A.A. (2013). The papillomavirus E2 proteins. Virology.

[34] Thierry F. (2009). Transcriptional regulation of the papillomavirus oncogenes by cellular and viral transcription factors in cervical carcinoma. Virology.

[35] Wang X., Meyers C., Wang H.K., Chow L.T., Zheng Z.M. (2011). Construction of a full transcription map of human papillomavirus type 18 during productive viral infection. J. Virol..

[36] Chen J., Xue Y., Poidinger M., Lim T., Chew S.H., Pang C.L., Abastado J.P., Thierry F. (2014). Mapping of HPV transcripts in four human cervical lesions using RNAseq suggests quantitative rearrangements during carcinogenic progression. Virology.

[37] Taguchi A., Nagasaka K., Kawana K., Hashimoto K., Kusumoto-Matsuo R., Plessy C., Thomas M., Nakamura H., Bonetti A., Oda K. (2015). Characterization of novel transcripts of human papillomavirus type 16 using cap analysis gene expression technology. J. Virol..

[38] Schmitt M., Dalstein V., Waterboer T., Clavel C., Gissmann L., Pawlita M. (2011). The HPV16 transcriptome in cervical lesions of different grades. Mol. Cell. Probes.

[39] Kanduc D. (2002). Translational regulation of human papillomavirus type 16 E7 mRNA by the peptide SEQIKA, shared by rabbit α_1_-globin and human cytokeratin 7. J. Virol..

[40] Favia G., Kanduc D., Lo Muzio L., Lucchese A., Serpico R. (2004). Possible association between HPV16 E7 protein level and cytokeratin 19. Int. J. Cancer.

[41] Herfs M., Longuespee R., Quick C.M., Roncarati P., Suarez-Carmona M., Hubert P., Lebeau A., Bruyere D., Mazzucchelli G., Smargiasso N. (2017). Proteomic signatures reveal a dualistic and clinically relevant classification of anal canal carcinoma. J. Pathol..

[42] Gorodeski G.I., Hopfer U., Liu C.C., Margles E. (2005). Estrogen acidifies vaginal pH by up-regulation of proton secretion via the apical membrane of vaginal-ectocervical epithelial cells. Endocrinology.

[43] Herfs M., Hubert P., Kholod N., Caberg J.H., Gilles C., Berx G., Savagner P., Boniver J., Delvenne P. (2008). Transforming growth factor-β1-mediated Slug and Snail transcription factor up-regulation reduces the density of Langerhans cells in epithelial metaplasia by affecting E-cadherin expression. Am. J. Pathol..

[44] Caberg J.H., Hubert P.M., Begon D.Y., Herfs M.F., Roncarati P.J., Boniver J.J., Delvenne P.O. (2008). Silencing of E7 oncogene restores functional E-cadherin expression in human papillomavirus 16-transformed keratinocytes. Carcinogenesis.

[45] Hubert P., Caberg J.H., Gilles C., Bousarghin L., Franzen-Detrooz E., Boniver J., Delvenne P. (2005). E-cadherin-dependent adhesion of dendritic and Langerhans cells to keratinocytes is defective in cervical human papillomavirus-associated (pre)neoplastic lesions. J. Pathol..

[46] Jiang A., Bloom O., Ono S., Cui W., Unternaehrer J., Jiang S., Whitney J.A., Connolly J., Banchereau J., Mellman I. (2007). Disruption of E-cadherin-mediated adhesion induces a functionally distinct pathway of dendritic cell maturation. Immunity.

[47] Mayumi N., Watanabe E., Norose Y., Watari E., Kawana S., Geijtenbeek T.B., Takahashi H. (2013). E-cadherin interactions are required for Langerhans cell differentiation. Eur. J. Immunol..

[48] Herfs M., Herman L., Hubert P., Minner F., Arafa M., Roncarati P., Henrotin Y., Boniver J., Delvenne P. (2009). High expression of PGE2 enzymatic pathways in cervical (pre)neoplastic lesions and functional consequences for antigen-presenting cells. Cancer Immunol. Immunother. CII.

[49] Demoulin S.A., Somja J., Duray A., Guenin S., Roncarati P., Delvenne P.O., Herfs M.F., Hubert P.M. (2015). Cervical (pre)neoplastic microenvironment promotes the emergence of tolerogenic dendritic cells via RANKL secretion. Oncoimmunology.

[50] Demoulin S., Herfs M., Somja J., Roncarati P., Delvenne P., Hubert P. (2015). HMGB1 secretion during cervical carcinogenesis promotes the acquisition of a tolerogenic functionality by plasmacytoid dendritic cells. Int. J. Cancer.

[51] Brown D.R., Schroeder J.M., Bryan J.T., Stoler M.H., Fife K.H. (1999). Detection of multiple human papillomavirus types in Condylomata acuminata lesions from otherwise healthy and immunosuppressed patients. J. Clin. Microbiol..

[52] Alves de Sousa N.L., Alves R.R., Martins M.R., Barros N.K., Ribeiro A.A., Zeferino L.C., Dufloth R.M., Rabelo-Santos S.H. (2012). Cytopathic effects of human papillomavirus infection and the severity of cervical intraepithelial neoplasia: A frequency study. Diagn. Cytopathol..

[53] Vrdoljak-Mozetic D., Krasevic M., Versa Ostojic D., Stemberger-Papic S., Rubesa-Mihaljevic R., Bubonja-Sonje M. (2015). HPV16 genotype, p16/Ki-67 dual staining and koilocytic morphology as potential predictors of the clinical outcome for cervical low-grade squamous intraepithelial lesions. Cytopathology.

[54] Ceballos K.M., Chapman W., Daya D., Julian J.A., Lytwyn A., McLachlin C.M., Elit L. (2008). Reproducibility of the histological diagnosis of cervical dysplasia among pathologists from 4 continents. Int. J. Gynecol. Pathol..

[55] Dalla Palma P., Giorgi Rossi P., Collina G., Buccoliero A.M., Ghiringhello B., Gilioli E., Onnis G.L., Aldovini D., Galanti G., Casadei G. (2009). The reproducibility of CIN diagnoses among different pathologists: Data from histology reviews from a multicenter randomized study. Am. J. Clin. Pathol..

[56] Herfs M., Crum C.P. (2013). Laboratory management of cervical intraepithelial neoplasia: Proposing a new paradigm. Adv. Anat. Pathol..

[57] Park J.J., Sun D., Quade B.J., Flynn C., Sheets E.E., Yang A., McKeon F., Crum C.P. (2000). Stratified mucin-producing intraepithelial lesions of the cervix: Adenosquamous or columnar cell neoplasia?. Am. J. Surg. Pathol..

[58] Crum C.P. (2000). Contemporary theories of cervical carcinogenesis: The virus, the host, and the stem cell. Mod. Pathol..

[59] Smotkin D., Berek J.S., Fu Y.S., Hacker N.F., Major F.J., Wettstein F.O. (1986). Human papillomavirus deoxyribonucleic acid in adenocarcinoma and adenosquamous carcinoma of the uterine cervix. Obstet. Gynecol..

[60] Witkiewicz A.K., Hecht J.L., Cviko A., McKeon F.D., Ince T.A., Crum C.P. (2005). Microglandular hyperplasia: A model for the de novo emergence and evolution of endocervical reserve cells. Hum. Pathol..

[61] Palefsky J.M., Giuliano A.R., Goldstone S., Moreira E.D., Aranda C., Jessen H., Hillman R., Ferris D., Coutlee F., Stoler M.H. (2011). HPV vaccine against anal HPV infection and anal intraepithelial neoplasia. N. Engl. J. Med..

[62] Lehtinen M., Paavonen J., Wheeler C.M., Jaisamrarn U., Garland S.M., Castellsague X., Skinner S.R., Apter D., Naud P., Salmeron J. (2012). Overall efficacy of HPV-16/18 AS04-adjuvanted vaccine against grade 3 or greater cervical intraepithelial neoplasia: 4-year end-of-study analysis of the randomised, double-blind PATRICIA trial. Lancet Oncol..

[63] Munoz N., Kjaer S.K., Sigurdsson K., Iversen O.E., Hernandez-Avila M., Wheeler C.M., Perez G., Brown D.R., Koutsky L.A., Tay E.H. (2010). Impact of human papillomavirus (HPV)-6/11/16/18 vaccine on all HPV-associated genital diseases in young women. J. Natl. Cancer Inst..

[64] Schiller J.T., Castellsague X., Garland S.M. (2012). A review of clinical trials of human papillomavirus prophylactic vaccines. Vaccine.

[65] De Sanjose S., Diaz M., Castellsague X., Clifford G., Bruni L., Munoz N., Bosch F.X. (2007). Worldwide prevalence and genotype distribution of cervical human papillomavirus DNA in women with normal cytology: A meta-analysis. Lancet Infect. Dis..

[66] Chesson H.W., Ekwueme D.U., Saraiya M., Watson M., Lowy D.R., Markowitz L.E. (2012). Estimates of the annual direct medical costs of the prevention and treatment of disease associated with human papillomavirus in the United States. Vaccine.

[67] Massad L.S., Einstein M.H., Huh W.K., Katki H.A., Kinney W.K., Schiffman M., Solomon D., Wentzensen N., Lawson H.W., Conference A.C.G. (2013). 2012 updated consensus guidelines for the management of abnormal cervical cancer screening tests and cancer precursors. Obstet. Gynecol..

[68] Sagasta A., Castillo P., Saco A., Torne A., Esteve R., Marimon L., Ordi J., Del Pino M. (2016). p16 staining has limited value in predicting the outcome of histological low-grade squamous intraepithelial lesions of the cervix. Mod. Pathol..

[69] Liao G.D., Sellors J.W., Sun H.K., Zhang X., Bao Y.P., Jeronimo J., Chen W., Zhao F.H., Song Y., Cao Z. (2014). p16INK4A immunohistochemical staining and predictive value for progression of cervical intraepithelial neoplasia grade 1: A prospective study in China. Int. J. Cancer.

[70] Matsumoto K., Oki A., Furuta R., Maeda H., Yasugi T., Takatsuka N., Mitsuhashi A., Fujii T., Hirai Y., Iwasaka T. (2011). Predicting the progression of cervical precursor lesions by human papillomavirus genotyping: A prospective cohort study. Int. J. Cancer.

[71] Pajtler M., Milicic-Juhas V., Milojkovic M., Topolovec Z., Curzik D., Mihaljevic I. (2010). Assessment of HPV DNA test value in management women with cytological findings of ASC-US, CIN1 and CIN2. Coll. Antropol..

[72] Gravitt P.E., Kovacic M.B., Herrero R., Schiffman M., Bratti C., Hildesheim A., Morales J., Alfaro M., Sherman M.E., Wacholder S. (2007). High load for most high risk human papillomavirus genotypes is associated with prevalent cervical cancer precursors but only HPV16 load predicts the development of incident disease. Int. J. Cancer.

[73] Dalstein V., Riethmuller D., Pretet J.L., Le Bail Carval K., Sautiere J.L., Carbillet J.P., Kantelip B., Schaal J.P., Mougin C. (2003). Persistence and load of high-risk HPV are predictors for development of high-grade cervical lesions: A longitudinal French cohort study. Int. J. Cancer.

[74] Hesselink A.T., Berkhof J., Heideman D.A., Bulkmans N.W., van Tellingen J.E., Meijer C.J., Snijders P.J. (2009). High-risk human papillomavirus DNA load in a population-based cervical screening cohort in relation to the detection of high-grade cervical intraepithelial neoplasia and cervical cancer. Int. J. Cancer.

[75] Xi L.F., Koutsky L.A., Castle P.E., Wheeler C.M., Galloway D.A., Mao C., Ho J., Kiviat N.B. (2009). Human papillomavirus type 18 DNA load and 2-year cumulative diagnoses of cervical intraepithelial neoplasia grades 2–3. J. Natl. Cancer Inst..

[76] Holowaty P., Miller A.B., Rohan T., To T. (1999). Natural history of dysplasia of the uterine cervix. J. Natl. Cancer Inst..

[77] Schlecht N.F., Platt R.W., Duarte-Franco E., Costa M.C., Sobrinho J.P., Prado J.C., Ferenczy A., Rohan T.E., Villa L.L., Franco E.L. (2003). Human papillomavirus infection and time to progression and regression of cervical intraepithelial neoplasia. J. Natl. Cancer Inst..

[78] Herfs M., Crum C.P. (2015). Cervical cancer: Squamocolumnar junction ablation—Tying up loose ends?. Nat. Rev. Clin. Oncol..

[79] Herfs M., Somja J., Howitt B.E., Suarez-Carmona M., Kustermans G., Hubert P., Doyen J., Goffin F., Kridelka F., Crum C.P. (2015). Unique recurrence patterns of cervical intraepithelial neoplasia after excision of the squamocolumnar junction. Int. J. Cancer.

[80] Franceschi S. (2015). Past and future of prophylactic ablation of the cervical squamocolumnar junction. Ecancermedicalscience.

[81] Younge P.A. (1957). Cancer of the uterine cervix; a preventable disease. Obstet. Gynecol..

[82] Peyton F.W., Peyton R.R., Anderson V.L., Pavnica P. (1978). The importance of cauterization to maintain a healthy cervix. Long-term study from a private gynecologic practice. Am. J. Obstet. Gynecol..

[83] Kauraniemi T., Rasanen-Virtanen U., Hakama M. (1978). Risk of cervical cancer among an electrocoagulated population. Am. J. Obstet. Gynecol..

[84] Taylor S., Wang C., Wright T.C., Denny L., Tsai W.Y., Kuhn L. (2010). Reduced acquisition and reactivation of human papillomavirus infections among older women treated with cryotherapy: Results from a randomized trial in South Africa. BMC Med..

[85] Kocken M., Helmerhorst T.J., Berkhof J., Louwers J.A., Nobbenhuis M.A., Bais A.G., Hogewoning C.J., Zaal A., Verheijen R.H., Snijders P.J. (2011). Risk of recurrent high-grade cervical intraepithelial neoplasia after successful treatment: A long-term multi-cohort study. Lancet Oncol..

[86] Herfs M., Hubert P., Moutschen M., Delvenne P. (2011). Mucosal junctions: Open doors to HPV and HIV infections?. Trends Microbiol..

[87] Auvert B., Lissouba P., Cutler E., Zarca K., Puren A., Taljaard D. (2010). Association of oncogenic and nononcogenic human papillomavirus with HIV incidence. J. Acquir. Immune Defic. Syndr..

[88] Lissouba P., Van de Perre P., Auvert B. (2013). Association of genital human papillomavirus infection with HIV acquisition: A systematic review and meta-analysis. Sex. Transm. Infect..

[89] Averbach S.H., Gravitt P.E., Nowak R.G., Celentano D.D., Dunbar M.S., Morrison C.S., Grimes B., Padian N.S. (2010). The association between cervical human papillomavirus infection and HIV acquisition among women in Zimbabwe. Aids.

[90] Smith-McCune K.K., Shiboski S., Chirenje M.Z., Magure T., Tuveson J., Ma Y., Da Costa M., Moscicki A.B., Palefsky J.M., Makunike-Mutasa R. (2010). Type-specific cervico-vaginal human papillomavirus infection increases risk of HIV acquisition independent of other sexually transmitted infections. PLoS ONE.

[91] Tommasino M. (2017). The biology of beta human papillomaviruses. Virus Res..

[92] Viarisio D., Mueller-Decker K., Kloz U., Aengeneyndt B., Kopp-Schneider A., Grone H.J., Gheit T., Flechtenmacher C., Gissmann L., Tommasino M. (2011). E6 and E7 from beta HPV38 cooperate with ultraviolet light in the development of actinic keratosis-like lesions and squamous cell carcinoma in mice. PLoS Pathog..

[93] Mirabello L., Yeager M., Cullen M., Boland J.F., Chen Z., Wentzensen N., Zhang X., Yu K., Yang Q., Mitchell J. (2016). HPV16 Sublineage Associations With Histology-Specific Cancer Risk Using HPV Whole-Genome Sequences in 3200 Women. J. Natl. Cancer Inst..

[94] Pirog E.C., Kleter B., Olgac S., Bobkiewicz P., Lindeman J., Quint W.G., Richart R.M., Isacson C. (2000). Prevalence of human papillomavirus DNA in different histological subtypes of cervical adenocarcinoma. Am. J. Pathol..

[95] Kajitani N., Satsuka A., Kawate A., Sakai H. (2012). Productive Lifecycle of Human Papillomaviruses that Depends Upon Squamous Epithelial Differentiation. Front. Microbiol..

